# Digitization and Health in Germany: Cross-sectional Nationwide Survey

**DOI:** 10.2196/32951

**Published:** 2021-11-22

**Authors:** Karina Karolina De Santis, Tina Jahnel, Elida Sina, Julian Wienert, Hajo Zeeb

**Affiliations:** 1 Department of Prevention and Evaluation Leibniz Institute for Prevention Research and Epidemiology-BIPS Bremen Germany; 2 Leibniz Science Campus Digital Public Health Bremen Germany; 3 Faculty 11; Human and Health Sciences University of Bremen Bremen Germany; 4 Department of Epidemiological Methods and Etiological Research Leibniz Institute for Prevention Research and Epidemiology-BIPS Bremen Germany; 5 Faculty of Social Sciences IU International University of Applied Sciences Bad Reichenhall Germany

**Keywords:** digital health, literacy, survey, attitude, usage, eHEALS, COVID-19, physical activity, general population, misinformation

## Abstract

**Background:**

Digital technologies are shaping medicine and public health.

**Objective:**

The aim of this study was to investigate the attitudes toward and the use of digital technologies for health-related purposes using a nationwide survey.

**Methods:**

We performed a cross-sectional study using a panel sample of internet users selected from the general population living in Germany. Responses to a survey with 28 items were collected using computer-assisted telephone interviews conducted in October 2020. The items were divided into four topics: (1) general attitudes toward digitization, (2) COVID-19 pandemic, (3) physical activity, and (4) perceived digital health (eHealth) literacy measured with the eHealth Literacy Scale (eHEALS; sum score of 8=lowest to 40=highest perceived eHealth literacy). The data were analyzed in IBM-SPSS24 using relative frequencies. Three univariate multiple regression analyses (linear or binary logistic) were performed to investigate the associations among the sociodemographic factors (age, gender, education, and household income) and digital technology use.

**Results:**

The participants included 1014 internet users (n=528, 52.07% women) aged 14 to 93 years (mean 54, SD 17). Among all participants, 66.47% (674/1014) completed up to tertiary (primary and secondary) education and 45.07% (457/1017) reported a household income of up to 3500 Euro/month (1 Euro=US $1.18). Over half (579/1014, 57.10%) reported having used digital technologies for health-related purposes. The majority (898/1014, 88.56%) noted that digitization will be important for therapy and health care, in the future. Only 25.64% (260/1014) reported interest in smartphone apps for health promotion/prevention and 42.70% (433/1014) downloaded the COVID-19 contact-tracing app. Although 52.47% (532/1014) reported that they come across inaccurate digital information on the COVID-19 pandemic, 78.01% (791/1014) were confident in their ability to recognize such inaccurate information. Among those who use digital technologies for moderate physical activity (n=220), 187 (85.0%) found such technologies easy to use and 140 (63.6%) reported using them regularly (at least once a week). Although the perceived eHealth literacy was high (eHEALS mean score 31 points, SD 6), less than half (43.10%, 400/928) were confident in using digital information for health decisions. The use of digital technologies for health was associated with higher household income (odds ratio [OR] 1.28, 95% CI 1.11-1.47). The use of digital technologies for physical activity was associated with younger age (OR 0.95, 95% CI 0.94-0.96) and more education (OR 1.22, 95% CI 1.01-1.46). A higher perceived eHealth literacy score was associated with younger age (*β*=–.22, *P*<.001), higher household income (*β*=.21, *P*<.001), and more education (*β*=.14, *P*<.001).

**Conclusions:**

Internet users in Germany expect that digitization will affect preventive and therapeutic health care in the future. The facilitators and barriers associated with the use of digital technologies for health warrant further research. A gap exists between high confidence in the perceived ability to evaluate digital information and low trust in internet-based information on the COVID-19 pandemic and health decisions.

## Introduction

The COVID-19 pandemic contributed to the development of new technologies and accelerated the digitization of various domains of daily lives worldwide. One such domain focuses on digital aspects of public health. Digital public health describes the entire field of development and application of digital technologies in the context of public health, especially with regard to prevention and health promotion [1]. So far, digital technologies have shown potential for innovation, particularly in the area of individual health promotion, the use of health apps for prevention and early disease diagnosis, as well as for health education [2]. Digital technologies are thus likely to influence health-related decisions in the future [3].

Coincidentally, shortly before the onset of the COVID-19 pandemic (in August 2019), an innovative project was launched in the city of Bremen in Northern Germany. Specifically, Leibniz-Science Campus Digital Public Health Bremen (LSC DiPH) was established as a virtual network linking three local institutions with expertise on digital technology in medicine and public health (the Leibniz Institute for Prevention Research and Epidemiology-BIPS, the University of Bremen, and the Fraunhofer Institute for Digital Medicine-MEVIS) [4]. The general aims of LSC DiPH are to provide a platform for networking and to support interdisciplinary projects in the field of digital public health, focusing on prevention and health promotion.

This study was designed within the scope of LSC DiPH. Our objective was to explore the attitudes toward digitization in the health context using a nationwide survey of internet users selected from the general population in Germany. Such user-driven attitudes and preferences are of particular interest at the time of the worldwide COVID-19 pandemic that contributed to digitization in the health context. We were especially interested in the central aspects of digital public health [1], including personal use of digital technologies for obtaining health information and for supporting prevention and health promotion. Our study aimed to explore four main topics related to digitization in the health context. First, we aimed to investigate the attitudes toward current and future applications of digital technologies for health-related purposes, privacy of data online, and preferences for smartphone apps addressing prevention and health promotion. Second, owing to the overabundance of information on the COVID-19 pandemic online [5], we were interested in how the general population evaluates such information. Third, owing to contact restrictions during the COVID-19 pandemic that reduced the (analog) offers for performing physical exercise [6], we aimed to assess the interest in and actual use of digital alternatives for supporting physical activity. Fourth, we aimed to assess the digital (eHealth) literacy that is an essential requirement of dealing with digital technologies for health-related purposes [7]. In general, eHealth literacy describes the ability to seek, find, understand, and evaluate health-related information online [7]. Finally, we were interested in exploring the question of who uses digital technologies in the health context. In general, it has been shown that the privileged members of the general population (wealthier, younger, and more educated) have more access to and may receive a greater benefit from digital technologies [8]. Thus, we aimed to investigate the associations among sociodemographic factors and digital technology use in the health context.

## Methods

### Study Design

We performed a nationwide, cross-sectional survey using a representative sample of 1014 internet users selected from the general population living in Germany. Detailed information on the recruitment strategy is provided in Multimedia Appendix 1, Textbox S1.

### Participants

The sample was recruited by the market research institute Kantar GmbH (Munich, Germany) from an existing panel. The participants were required to be internet users, aged 14 years or older, live in Germany, and able to complete the interview in German. Sociodemographic variables (age; gender; education; employment; household size [number of members]; household income; and residence by population size, region, and state) were collected to ensure that the sample was representative of the general population of Germany according to data from the Federal Statistical Office and the Microcensus. Ethical permission to perform the study was not required because the authors had no contact with the participants and obtained fully anonymized data from Kantar GmbH.

### Procedure

The data were collected by Kantar GmbH using computer-assisted telephone interviews in October 2020. The participants were contacted by telephone using a random-digit-dial method. A dual-frame approach was used to ensure that both landline and mobile-only users were included. Each interview lasted about 15 minutes and was conducted in German.

### Survey Items

**Figure 1:**
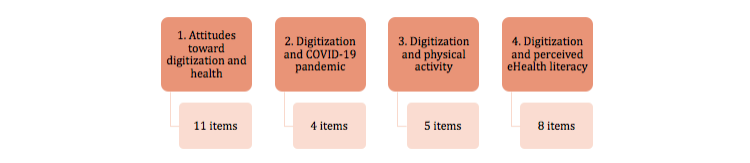
Survey with 28 items divided into four topics.

The interviews were conducted using a survey with 28 items divided into four topics (Figure 1).

All 28 items and answer options are reported in Multimedia Appendix 1, Table S1. The items were selected from existing and validated instruments in English or in German, which were adapted to the four topics and/or translated to German if translation was not available (see Multimedia Appendix 2). Perceived eHealth literacy was measured with the German version of the eHealth Literacy Scale (eHEALS) [9]. eHEALS consists of eight items with statements that address the ability to locate, evaluate, and use internet-based resources for health-related purposes [10]. The items are rated on a 5-point Likert Scale (from 1=strongly disagree to 5=strongly agree). The overall sum score indicates the level of perceived eHealth literacy (from 8=lowest to 40=highest). eHEALS has acceptable psychometric properties [9,10].

### Statistical Analysis

The statistical analysis was performed in IBM-SPSS24. First, the responses on all 28 items were analyzed using relative frequencies per item. This analysis was performed on raw responses unweighted by the sociodemographic factors because the survey data included some missing responses due to optional items. Specifically, the survey consisted of 13/28 mandatory items and 2/28 filter items that determined if the subsequent 5/28 optional items were asked or omitted. Furthermore, 8/28 eHEALS items had to be completely rated to compute the overall perceived eHealth literacy score for each participant and thus incomplete data had to be excluded from the analysis. To investigate the impact of the sociodemographic factors, we visually compared the responses on 13 mandatory items unweighted or weighted by the sociodemographic factors and report the weighted frequencies in Multimedia Appendix 1. Second, Cronbach α was computed to test the internal consistency of the unweighted responses on all eight eHEALS items. Third, three univariate multiple regression analyses (linear or binary logistic) were performed to investigate the associations among the sociodemographic factors and digital technology use. Each regression analysis included one dependent variable (the use of digital technologies for health or physical activity, or the perceived eHealth literacy score) and four independent variables (sociodemographic factors: age, gender, education, and household income). Variable coding and further details of these analyses are reported in Multimedia Appendix 1.

## Results

### Participants

**Figure 2:**
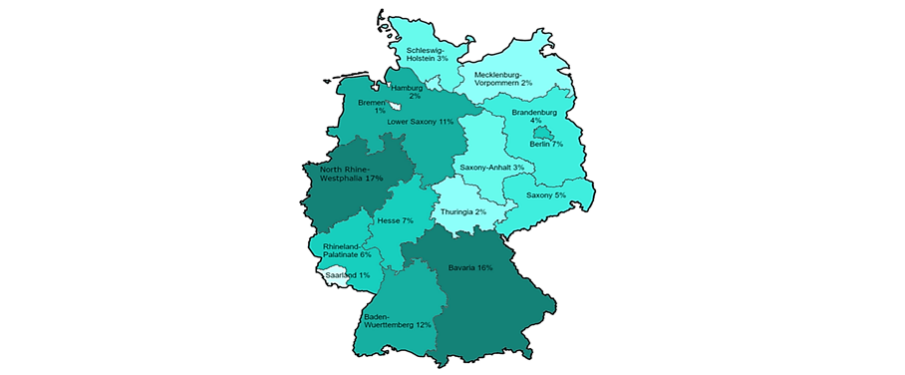
Participant location by state in Germany.

The data from 1014 internet users were obtained via either landline (n=826, 81.46%) or mobile (n=188, 18.54%) telephones. The participants were recruited from all 16 states in Germany (Figure 2) with the majority residing in urban regions with up to 500,000 inhabitants (622/1014, 61.34%) and in the states of the former West Germany (829/1014, 81.76%; Multimedia Appendix 1, Table S2).

The sociodemographic characteristics of the 1014 participants are reported in Table 1. The participants (52% women) were aged 14 to 93 years (mean 54, SD 17). Of those, 66% completed up to tertiary (primary and secondary) education, 60% were either employed or seeking employment, 67% lived in 1-2–person households, and 45% reported a net household income of up to 3500 Euro/month (1 Euro=US $1.18).

**Table 1:** Participant sociodemographic characteristics (N=1014).

Variable^a^		Participants n (%)
**Gender**		
	Female	528 (52.07)
	Male	486 (48.03)
**Education**		
	Elementary/primary school	17 (1.68)
	Vocational college or basic secondary	101 (9.96)
	Secondary without tertiary entrance qualification	269 (26.53)
	Secondary with tertiary entrance qualification	287 (28.30)
	Tertiary	340 (33.53)
**Employed**		
	Yes or seeking employment	607 (59.86)
	No	407 (40.14)
**Household size (members)**		
	1	239 (23.57)
	2	436 (43.00)
	3	162 (15.98)
	4	123 (12.13)
	5 or more	54 (5.33)
**Household net income/month (Euro)^b^**		
	under 1500	94 (9.27)
	1500 up to 2500	171 (16.86)
	2500 up to 3500	192 (18.93)
	3500 or more	370 (36.49)
	no response	187 (18.44)

^a^Further characteristics are shown in Multimedia Appendix 1, Table S2.

^b^1 Euro=US $1.18 in 2020; the mean net household income in Germany was 3580 Euro/month in 2019-2020 [11].

### Attitudes Toward Digitization and Health

**Figure 3:**
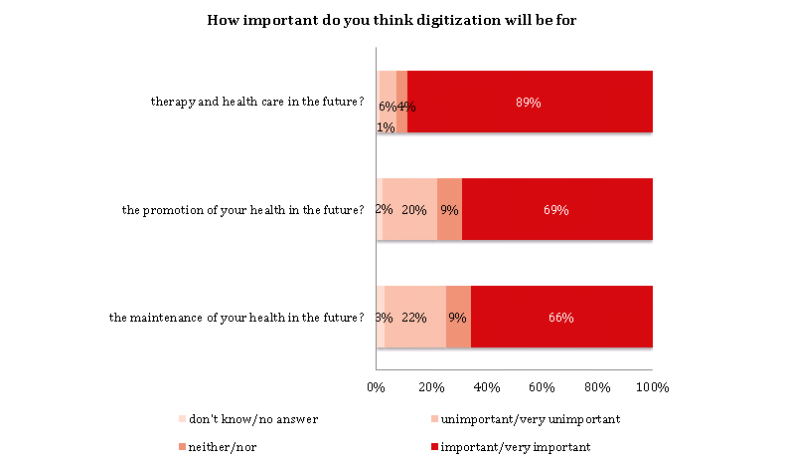
Digitization of health in the future (N=1014).

Over half of the participants (579/1014, 57.10%) reported having used digital technologies for health-related purposes. The majority noted that digitization will be important for therapy and health care (898/1014, 88.56%), health promotion (704/1014, 69.43%), and health maintenance (668/1014, 65.88%) in the future (Figure 3; Multimedia Appendix 1, Table S3 and Figure S1).

**Figure 4:**
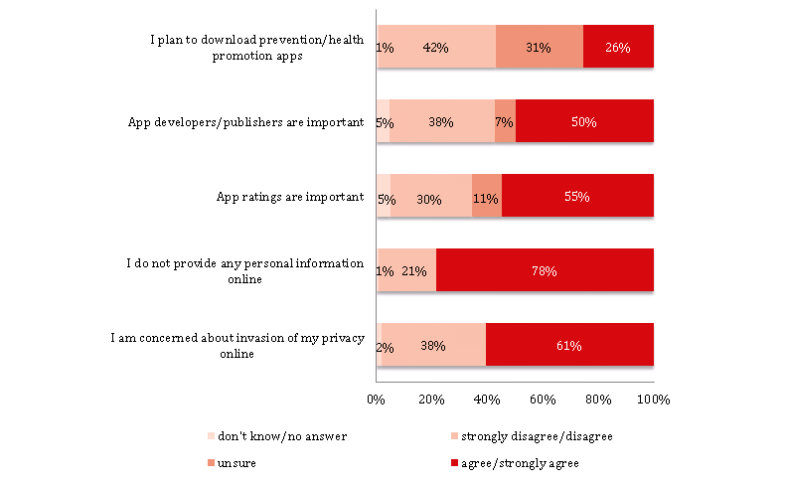
Digitization, smartphone apps, and internet use (N=1014).

When asked about smartphone apps, 25.64% (260/1014) planned to download prevention/health promotion apps (Figure 4; Multimedia Appendix 1, Table S4 and Figure S2). The choice of apps depended on developers/publishers (507/1014, 55.00%) or ratings (558/1014, 55.03%). In terms of general internet use, 78.40% (795/1014) reported that they do not provide any personal information online and 60.55% (614/1014) were concerned about the invasion of their privacy online (Figure 4). The majority reported using social media platforms (868/1014, 85.60%). Among those who use social media (n=868), 593 (68.32%) typically access these platforms up to 10 times per day and 560 (64.52%) prefer messaging platforms such as Facebook Messenger, Viber, or WhatsApp (Multimedia Appendix 1, Table S4).

### Digitization and the COVID-19 Pandemic

**Figure 5:**
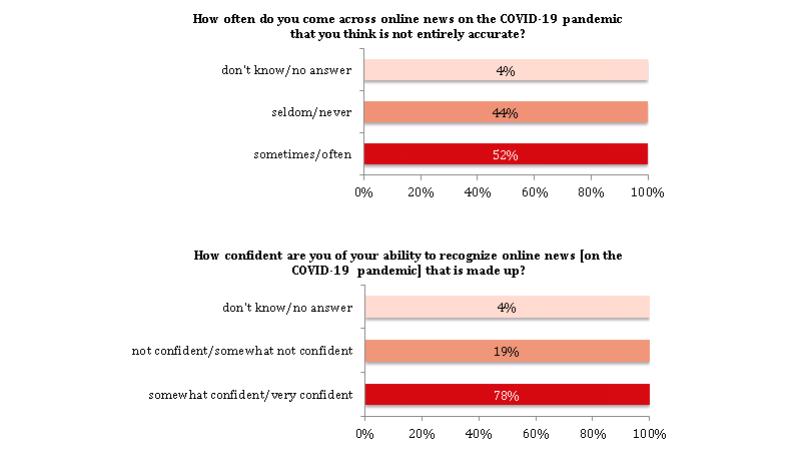
Digitization and COVID-19 pandemic (N=1014).

Approximately half of the participants (532/1014, 52.47%) thought that the online news on the COVID-19 pandemic is, in some cases, not entirely accurate and the majority reported that they were confident in their ability to recognize such false online news (791/1014, 78.01%; Figure 5; Multimedia Appendix 1, Table S5 and Figure S3). Only a minority reported having shared false online news on the COVID-19 pandemic (56/1014, 5.52%) and 42.70% (433/1014) installed the contact-tracing app from the Robert Koch Institute in Germany by October 2020.

### Digitization and Physical Activity

**Figure 6:**
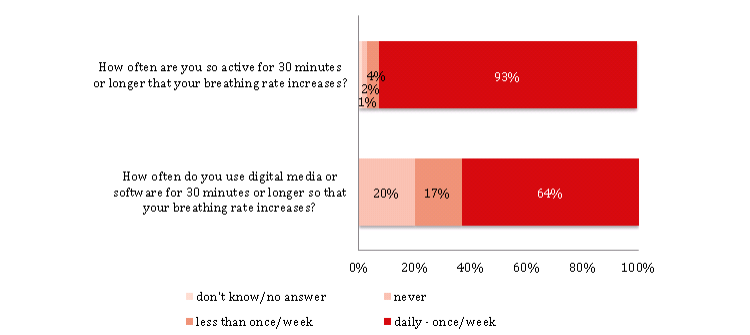
Digitization and physical activity (n=220).

Although less than a quarter of the participants (220/1014, 21.70%) reported having used digital technologies to support moderate physical activity (ie, physical activity that leads to an increase in the breathing rate), most of these users (187/220, 85.0%) found such technologies easy to use (Multimedia Appendix 1, Table S6). In addition, most of these users (204/220, 92.73%) also reported that they regularly participate in moderate physical activity for 30 minutes or longer at least once a week and use digital technologies for such regular physical activity (140/220, 63.64%; Figure 6).

### Digitization and Perceived eHealth Literacy (eHEALS)

**Figure 7:**
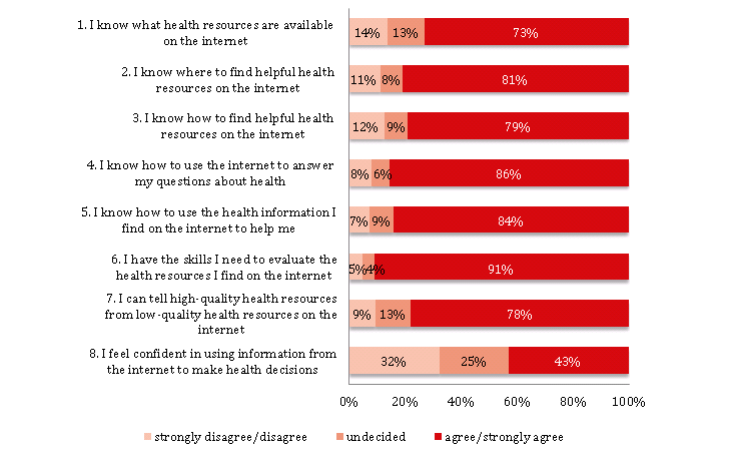
Digitization and perceived eHealth literacy (eHEALS; n=928).

Complete responses on all eight items of the eHEALS were provided by 928 participants. The internal consistency of the responses was high (Cronbach α=.88; Multimedia Appendix 1, Table S7). The perceived eHealth literacy was high in our sample (eHEALS mean 31 points, SD 6; Multimedia Appendix 1, Table S8). Responses on eHEALS items 1 to 7 indicated that most participants (73%-91%) reported being able to locate, find, use, and evaluate health-related information online (Figure 7; Multimedia Appendix 1, Table S9). However, responses on eHEALS item 8 indicated that only 43.10% (400/928) were confident in using such online information for health-related decisions (Figure 7).

### Sociodemographic Factors and Digital Technology Use

Three univariate multiple regression analyses were performed to investigate the associations among the sociodemographic factors and digital technology use. Participants who did not report their household income were excluded from each analysis. Odds ratios (ORs) with 95% CIs were computed using bivariate logistic regression analyses; *β* coefficients were computed using univariate multiple linear regression analysis. Variable coding and further details of these analyses are reported in Multimedia Appendix 1, Table S10.

**Figure 8:**
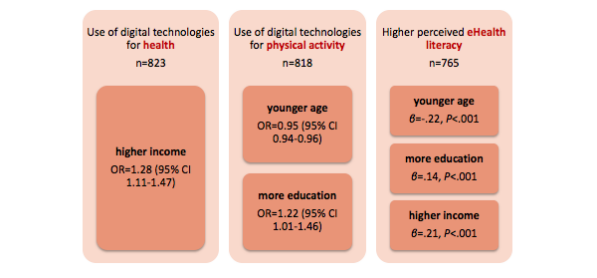
Associations among sociodemographic factors and digital technology use. OR: odds ratio.

All three regression analyses showed that the sociodemographic factors (age, education, and household income) were associated with digital technology use (Figure 8). The use of digital technologies for health was associated with higher income. The use of digital technologies for physical activity was associated with younger age and more education. Higher perceived eHealth literacy was associated with younger age, higher income, and more education.

## Discussion

### Principal Results

This exploratory study reports a snapshot of attitudes toward digitization in the health context among internet users selected from the general population in Germany. The vast majority of our participants expected that digitization will affect health care and central aspects of public health (prevention and health promotion) in Germany. However, the interest in and actual use of digital technologies for health-related purposes was not yet widespread in late 2020. The users of digital technologies for physical activity found such technologies easy to use and actually used them regularly. The confidence in the ability to recognize false online news and the perceived eHealth literacy were high. However, the trust in online information on the COVID-19 pandemic and the confidence in internet-based health decisions were low. Younger, more educated, and wealthier participants were more likely to use digital technologies for health-related purposes and reported higher eHealth literacy. The main strength of our study is the large, representative sample (N=1014) with a wide age range from adolescence to late adulthood (14 to 93 years) selected from the general population in Germany. Thus, we were able to explore the attitudes toward digitization in the health context across different life stages. Some of our results confirm what is generally known about the users of digital technologies who tend to be more privileged (wealthier, more educated, and more digitally literate) [8]. However, our study also provides novel, interesting, and partially unexpected results that warrant further research. These include the low trust in health information online despite the high perceived eHealth literacy and the low interest in and prevalence of digital technology use for health promotion, such as for physical exercise.

### Digitization and Health: Promises and Challenges

Widespread internet access and, in particular, the introduction of smartphones and other mobile devices since about 2009 have greatly contributed to the digitization of health [12]. Among others, digitization affects services, increases the availability of information, simplifies communication, and allows individual monitoring and self-measurement [1,13]. Indeed, our participants expected that especially therapy and health care services will be affected by digitization in the future. They also anticipated that digital technologies will be important to facilitate disease prevention and health promotion in the future. These could be achieved with the objective recording of data in everyday settings using increasingly affordable and user-friendly devices and digital apps [2]. Such data simplify monitoring of health and health-related behavior in daily life, and could positively reinforce behavioral patterns that contribute to a healthy lifestyle, including physical activity and nutrition [14]. In the long term, such data could be used for clinical decision-making [1]. However, rapid technological advancement is associated with several challenges on individual-, social-, and care-related levels, such as the ethical and legal aspects of data acquisition, storage, and application [1,13,15,16]. Aligned with these challenges, the majority of our participants expressed concern about their personal data and privacy online. Other issues such as the analog social environment, financial barriers, and digital competence and literacy [16] should also be considered in the future research on digitization and health. Furthermore, amid the ever-growing number of digital technology offers, in particular health-related apps, regulation is required in relation to safety, quality (ie, evidence-based content), and data protection (eg, introducing a quality score or label within the app store) to facilitate health decision-making among users of these offers [17,18].

### Digital Technology Use

Digital technologies show great innovation potential, especially in the area of individual health promotion as well as in relation to health education [2]. However, less than 50% of our participants showed interest in smartphone apps for disease prevention and health promotion, the COVID-19 contact-tracing app, or digital technologies for physical activity in late 2020. This is surprising, because during the COVID-19 pandemic, the use of digital technologies for health-related purposes increased rapidly worldwide [15]. We can only speculate that the results of our study were affected by the timing of data collection that aligned with relaxing of contact restrictions in Germany between the European summer and late November 2020. Since various health-related activities, including organized sport and educational offers, were allowed “in person” at that time, the participants might have embraced such an analog lifestyle by showing less interest in digital technologies. However, it is possible that many returned to or started using digital technologies during the subsequent tightening of contact restrictions that followed in Germany during the European winter 2020 until summer 2021. Furthermore, preferences for apps addressing physical activity could also depend on other factors such as age. For example, a focus group study in Germany showed that older adults prefer to use simple-to-use fitness apps with few features, automated tracking of data, and active feedback to reach their goals [19]. A repetition of the present survey could be used to quantitatively investigate the influence of the COVID-19 pandemic and other factors on the use of digital technologies for physical activity promotion. Qualitative methods could be used to explore the more in-depth reasons that encourage or hinder the use of digital technologies for physical activity promotion depending on the sociodemographic characteristics of users.

Although used by the minority, the users reported that digital technologies were easy and frequently utilized for moderate physical exercise. Although digital technologies for physical exercise are already accepted [20], their development and effectiveness require systematic evaluation [1,14,20]. For example, effectiveness of digital technologies for physical activity promotion depends on engagement with such technologies [21]. In general, digital technologies could be useful at improving education (health literacy) on lifestyle-related disorders [22] and at fostering positive health behavior changes [14]. However, it remains unclear why only some digitally based health interventions work [23] and how such interventions support behavior change, including healthy lifestyle promotion, in real-world settings. Thus, evaluation studies with large samples are necessary to examine the effects of digital technologies on various aspects of health, including education, promotion of healthy lifestyle, and prevention. The focus of such studies could be on the clinical effectiveness of interventions with modern (digital) technologies relative to the traditional (analog) health interventions. However, social, economic, or ecological factors should also be investigated to understand the impact of digital technologies on health. In addition, the research and application of new study designs and methods for evaluation purposes is necessary to take into account the rapid development of digital technologies and continuous data collection.

### Trust in Online Information and eHealth Literacy

Our data suggest that the general population in Germany used the internet for health education purposes (ie, to obtain information on the COVID-19 pandemic). However, the trust in such online information was low in our sample. This low trust could be associated with the so-called “infodemic” or abundance of correct and invented information on the COVID-19 pandemic available online [5]. The trust in online information also depends on digital competence and digital (eHealth) literacy that are important prerequisites for dealing with digital technologies for health-related purposes [5,24]. For example, those with low eHealth literacy have difficulties in recognizing invented, nonfactual information online [5]. According to an anonymous online survey conducted during the COVID-19 pandemic in late 2020 in Germany, approximately half of the 8500 participants selected from the general adult population reported limited eHealth literacy skills, in particular in terms of searching for and evaluating the reliability and relevance of health-related information online [24]. These findings closely align with results of another anonymous online survey of nearly 15,000 university students conducted in early 2020 in Germany [25]. Regardless of their high education status, the university students also reported difficulties with specific aspects of evaluation such as assessing the reliability and the commercial interests in the online information on the COVID-19 pandemic [25]. In contrast to these studies, our sample appeared to be more confident in their ability to evaluate the health resources online and reported a generally high level of perceived eHealth literacy. Such possible overestimation of eHealth literacy in our sample could be due to the different methods of data collection and tools utilized in different studies.

First, our results could be inflated by social desirability bias since our data were collected using computer-assisted telephone interviews rather than anonymous online surveys that were conducted in the other two studies in Germany [24,25]. Second, eHealth literacy was measured with the Digital Health Literacy Instrument in the other studies [24,25] and with eHEALS [9] in our study. Our data revealed that the responses on the eHEALS were highly consistent with the Cronbach α of .88 but also contradictory in some aspects: although many participants perceived their own eHealth literacy as high, less than 50% were confident in making health-related decisions based on information from the internet. Other studies that utilized eHEALS also reported similar Cronbach α coefficients [9,26] and high perceived eHealth literacy [9,26,27]. Similar to our results, the majority of participants in one of the studies [9] thought they had the skills to critically evaluate information online but only a minority felt confident in making health decisions based on such information. Interestingly, subjective (self-reported and perception-based) estimation of eHealth literacy was not associated with accurate judgments of the quality of a medical website or behavioral intentions beneficial to health [28]. Thus, high perceived eHealth literacy may be insufficient for making real-life decisions. Factors that promote or hinder behaviors and concrete actions related to eHealth literacy should be examined in further studies [29]. Furthermore, the reasons for low confidence in internet-based information and health decisions could be examined qualitatively. For example, internet users could report how they rate the health-related information on the internet, under what conditions they trust such information, and what factors would assist them with decision-making. Such qualitative data could then be used to design specific measures for evaluating online information with and for the general population in the context of participatory research.

### Digital Divide

The use of digital technologies in the health context is associated with various ethical, legal, and social issues [13,30]. Our results confirm that privileged people (wealthier, younger, and more educated) tend to be more digitally literate and are more likely to use digital technologies for health-related purposes. These findings support the notion that a “digital divide” or the promotion of inequality via digital technologies is present in the health care context in Germany, similar to reports from other countries [8]. A debate regarding digital inequalities is not new [31,32] and various sociodemographic factors associated with digital technology use have already been identified in the health context [8]. The digital divide has become especially evident in the context of the COVID-19 pandemic that rapidly digitized health care [33]. Factors that continue to contribute to digital inequalities include poor internet access, low experience with and variable expectations toward digital health care, low digital literacy and technological skills of health care users and providers, inadequate means to purchase tools at a time of high economic instability, and a gap between digital health care offers and patient capability to access and effectively utilize such offers [33]. Since privileged people may disproportionately benefit from the advantages of digital technologies [13], interventions that address the digital divide should be designed to specifically target less privileged groups [32]. This is important to reduce the digital divide to better align access to and outcomes of digital health care, especially for the most vulnerable groups.

### Limitations

There were several limitations of this study. First, the data were collected using a single source (quantitative survey) and relied on self-reports. Although the instrument was not validated, the survey items were selected from other existing, validated instruments and adapted to the purposes of this study. Second, there were very few items per topic, meaning that we were unable to gain detailed insight into the motivations for or against use and the types of digital technologies used for specific health-related purposes. Third, we did not weigh all data according to the sociodemographic factors due to the missing values on the optional items. However, weighing of responses on 13 mandatory items produced similar results to unweighted responses on these items (Multimedia Appendix 1, Figures S1-S3). Fourth, the associations among the sociodemographic factors and digital technology use for health-related purposes were relatively weak according to the univariate regressions. It is likely that such associations are complex and depend on further factors and/or on the interactions among multiple factors. Moreover, due to the cross-sectional design of our study, we were unable to investigate the causality in the associations among sociodemographic factors and digital technology use. Longitudinal studies with adequate follow-up are warranted to investigate how sociodemographic factors affect digital technology use.

### Conclusions

Internet users in Germany expect that digitization will affect health care in the future. However, the interest in and actual use of digital technologies for health-related purposes was relatively low in Germany in late 2020. The use of digital technologies is generally accepted for some purposes such as for physical activity promotion, but depends on age, household income, and education. Despite the high perceived eHealth literacy, the trust in online information and in health decisions based on such information is low, as exemplified by the COVID-19 pandemic. Thus, there is a need to study the reasons for the low trust and the high confidence in the ability to evaluate health information online. Further research should also address the needs, preferences, and motivations of users to identify facilitators and barriers associated with digital technology use for health-related purposes.

## Appendix

Multimedia Appendix 1 Survey items, additional data, and results (Textbox S1, Tables S1-S10, Figures S1-S3).

Multimedia Appendix 2 Survey item sources.

## Abbreviations

eHEALS: eHealth Literacy Scale
LSC DiPH: Leibniz-Science Campus Digital Public Health Bremen
OR: odds ratio

## References

[1] Zeeb H, Pigeot I, Schüz B, Leibniz-Wissenschafts Campus Digital Public Health Bremen (2020). Digital public health-an overview. Bundesgesundheitsblatt Gesundheitsforschung Gesundheitsschutz.

[2] Dadaczynski K, Tolks D (2018). Digital public health: opportunities and challenges of internet-based technologies and applications. Public Health Forum.

[3] Odone A, Buttigieg S, Ricciardi W, Azzopardi-Muscat N, Staines A (2019). Public health digitalization in Europe. Eur J Public Health.

[4] Leibniz-Science Campus Digital Public Health Bremen (LSC DiPH).

[5] Okan O, Bollweg TM, Berens E, Hurrelmann K, Bauer U, Schaeffer D (2020). Coronavirus-related health literacy: a cross-sectional study in adults during the COVID-19 infodemic in Germany. Int J Environ Res Public Health.

[6] Tison GH, Avram R, Kuhar P, Abreau S, Marcus GM, Pletcher MJ, Olgin JE (2020). Worldwide effect of COVID-19 on physical activity: a descriptive study. Ann Intern Med.

[7] Norman CD, Skinner HA (2006). eHealth literacy: essential skills for consumer health in a networked world. J Med Internet Res.

[8] Cornejo Müller A, Wachtler B, Lampert T (2020). Digital divide-social inequalities in the utilisation of digital healthcare. Bundesgesundheitsblatt Gesundheitsforschung Gesundheitsschutz.

[9] Soellner R, Huber S, Reder M (2014). The concept of eHealth literacy and its measurement. J Media Psychol.

[10] Norman CD, Skinner HA (2006). eHEALS: The eHealth Literacy Scale. J Med Internet Res.

[11] Income, receipts, expenditure (2019). Statistisches Bundesamt (Destatis).

[12] Taj F, Klein MCA, van Halteren A (2019). Digital health behavior change technology: bibliometric and scoping review of two decades of research. JMIR Mhealth Uhealth.

[13] Marckmann G (2020). Ethical implications of digital public health. Bundesgesundheitsblatt Gesundheitsforschung Gesundheitsschutz.

[14] Aromatario O, Van Hoye A, Vuillemin A, Foucaut A, Crozet C, Pommier J, Cambon L (2019). How do mobile health applications support behaviour changes? A scoping review of mobile health applications relating to physical activity and eating behaviours. Public Health.

[15] Gómez-Ramírez O, Iyamu I, Ablona A, Watt S, Xu AXT, Chang H, Gilbert M (2021). On the imperative of thinking through the ethical, health equity, and social justice possibilities and limits of digital technologies in public health. Can J Public Health.

[16] Schüz B, Urban M (2020). Unintended consequences and side effects of digital health technology: a public health perspective. Bundesgesundheitsblatt Gesundheitsforschung Gesundheitsschutz.

[17] Paganini S, Terhorst Y, Sander LB, Catic S, Balci S, Küchler AM, Schultchen D, Plaumann K, Sturmbauer S, Krämer LV, Lin J, Wurst R, Pryss R, Baumeister H, Messner E (2021). Quality of physical activity apps: systematic search in app stores and content analysis. JMIR Mhealth Uhealth.

[18] Fallaize R, Zenun Franco R, Pasang J, Hwang F, Lovegrove JA (2019). Popular nutrition-related mobile apps: an agreement assessment against a UK reference method. JMIR Mhealth Uhealth.

[19] Wichmann F, Sill J, Hassenstein MJ, Zeeb H, Pischke CR (2018). Apps zur Förderung von körperlicher Aktivität. Präv Gesundheitsf.

[20] Fischer F (2020). Digital interventions in prevention and health promotion: what kind of evidence do we have and what is needed?. Bundesgesundheitsblatt Gesundheitsforschung Gesundheitsschutz.

[21] Mclaughlin M, Delaney T, Hall A, Byaruhanga J, Mackie P, Grady A, Reilly K, Campbell E, Sutherland R, Wiggers J, Wolfenden L (2021). Associations between digital health intervention engagement, physical activity, and sedentary behavior: systematic review and meta-analysis. J Med Internet Res.

[22] Aida A, Svensson T, Svensson AK, Chung U, Yamauchi T (2020). eHealth delivery of educational content using selected visual methods to improve health literacy on lifestyle-related diseases: literature review. JMIR Mhealth Uhealth.

[23] Sporrel K, Nibbeling N, Wang S, Ettema D, Simons M (2021). Unraveling mobile health exercise interventions for adults: scoping review on the implementations and designs of persuasive strategies. JMIR Mhealth Uhealth.

[24] Kolpatzik K, Mohrmann M, Zeeb H (2020). Digital health competence in Germany. AOK Die Gesundheitskasse.

[25] Dadaczynski K, Okan O, Messer M, Leung AYM, Rosário R, Darlington E, Rathmann K (2021). Digital health literacy and web-based information-seeking behaviors of university students in Germany during the COVID-19 pandemic: cross-sectional survey study. J Med Internet Res.

[26] Holch P, Marwood JR (2020). EHealth literacy in UK teenagers and young adults: exploration of predictors and factor structure of the eHealth Literacy Scale (eHEALS). JMIR Form Res.

[27] Juvalta S, Kerry MJ, Jaks R, Baumann I, Dratva J (2020). Electronic Health Literacy in Swiss-German parents: cross-sectional study of eHealth Literacy Scale unidimensionality. J Med Internet Res.

[28] Schulz PJ, Pessina A, Hartung U, Petrocchi S (2021). Effects of objective and subjective health literacy on patients' accurate judgment of health information and decision-making ability: survey study. J Med Internet Res.

[29] Okan O, de Sombre S, Hurrelmann K, Berens E, Bauer U, Schaeffer D (2020). COVID-19 based health literacy in the German population. Monitor Versorgungsforschung.

[30] Cordeiro JV (2021). Digital technologies and data science as health enablers: an outline of appealing promises and compelling ethical, legal, and social challenges. Front Med (Lausanne).

[31] Robinson L, Cotten SR, Ono H, Quan-Haase A, Mesch G, Chen W, Schulz J, Hale TM, Stern MJ (2015). Digital inequalities and why they matter. Inf Commun Soc.

[32] Vassilakopoulou P, Hustad E (2021). Bridging digital divides: a literature review and research agenda for information systems research. Inf Syst Front.

[33] Ramsetty A, Adams C (2020). Impact of the digital divide in the age of COVID-19. J Am Med Inform Assoc.

[34] De Santis KK, Jahnel T, Sina E, Wienert J, Zeeb H (2021). Digitization and health: Results of a nationwide survey in Germany. Leibniz Institute for Prevention Research and Epidemiology-BIPS.

