# Supplementary material for: Digitization and Health in Germany: Cross-sectional Nationwide Survey
Source: JMIR Public Health Surveill. 2021 Nov 22;7(11):e32951. doi: 10.2196/32951 (PMC8612128; doi:10.2196/32951)
Supplement: Multimedia Appendix 1 [file publichealth_v7i11e32951_app1.docx]

Multimedia Appendix 1

**Digitization and Health in Germany: Results of a Nationwide Survey**

Content

[Textbox S1. Recruitment strategy 2](#_Toc87174749)

[Table S1. Survey items 3](#_Toc87174750)

[Table S2. Participant characteristics (N=1014) 7](#_Toc87174751)

[Table S3. Digitization of health in the future (N=1014) 8](#_Toc87174752)

[Table S4. Digitization, smartphone apps and Internet use (N=1014) 9](#_Toc87174753)

[Table S5. Digitization and COVID-19 pandemic (N=1014) 11](#_Toc87174754)

[Table S6. Digitization and physical activity (n=220) 12](#_Toc87174755)

[Table S7. Digitization and perceived eHealth literacy: internal consistency (n=928) 14](#_Toc87174756)

[Table S8. Digitization and perceived eHealth literacy: total sum score (n=928) 15](#_Toc87174757)

[Table S9. Digitization and perceived eHealth literacy: item statistics (n=928) 16](#_Toc87174758)

[Table S10. Sociodemographic factors and digital technology use 18](#_Toc87174759)

[Figure S1. Digitization of health in the future (N=1014; data weighted by region density, state, age, gender, employment, education and household size) 19](#_Toc87174760)

[Figure S2. Digitization, smartphone apps and Internet use (N=1014; data weighted by region density, state, age, gender, employment, education and household size) 20](#_Toc87174761)

[Figure S3. Digitization and COVID-19 pandemic (N=1014; data weighted by region density, state, age, gender, employment, education and household size) 21](#_Toc87174762)

## Textbox S1. Recruitment strategy

**Recruitement**

Using a random digit dialing design the landline sample was based on the Infratest Telephone Master Sample (ITMS). The ITMS is a household sample with a random selection of members in the household ensuring collection of a multi-stratified household sample designed to avoid cluster effects. Prior to selection, all phone numbers had to be assigned to a region in order to achieve a representative population density across geographical regions in the total sample. The telephone numbers were then randomly drawn for each municipality. Non-private numbers, numbers that have already been drawn and blocked numbers were not further considered for inclusion in the total sample. The multi-stratification and distribution of the sample to the cells took place fully automatically via an allocation program. The households that could not be reached were contacted up to ten times on different days of the week and at different times of the day. In case there were several members of the population in a household, one member was selected at random. If the target person was not present, an appointment was made for a repeat call. On the basis of the telephone number master list of the Federal Network Agency, all theoretically assigned mobile phone numbers were created for each mobile network. In order to avoid an overestimation, only numbers were generated from blocks of telephone numbers that actually contain entries (telephone book or Internet) and that were not reserved for technical services or special services. Since the phone numbers in the mobile phone sample cannot be located regionally in advance, the mobile phone sample was collected first. After each mobile phone interview has been completed, the individual data set was assigned to a regional cell according to the information provided by the interviewee. The landline sample for which telephone numbers can be selected based on their geographical region was collected last. The rules for contact attempts applied analogously to the landline sample. However, no further target person selection took place within the scope of the mobile phone sample: whoever was reached on the mobile phone was considered the target person.

**Sample size and weighting**

The sample size enabled a differentiated evaluation of the survey data according to individual socio-demographic (e.g., age, gender, education, employment) or regional (e.g., west-east, urban-rural) subgroups on a sufficient case basis with an acceptable statistical error tolerance.

The structural deviations in the results that usually occur with random samples are balanced out by means of factorial weighting. The weighting ensures that the sample corresponds in its composition to the structure of the population - according to age, gender and education. The weighting thus contributes to the representativeness of the total sample. The sample was weighted using population parameters from the current Federal Statistical Office and the Microcensus.

## Table S1. Survey items

| **No.** | **Item (German)** | **Answer option (German)** | **Item (English)** | **Answer option (English)** |
| --- | --- | --- | --- | --- |
| 1 | Nutzen Sie digitale Technologien für Ihre Gesundheit, z.B. zur persönlichen Einschätzung gesundheitlicher Risiken, zur Information über bestimmte Erkrankungen oder zur Unterstützung von Bewegung und Fitness? | 1 = ja; 2 = nein | Do you use digital technologies for your health, e.g. for personal assessment of health risks, for information about certain illnesses or to support exercise and fitness? | 1 = yes; 2 = no |
| 2 | Wie wichtig, glauben Sie, wird die Digitalisierung in der Zukunft für die Therapie und Gesundheitsversorgung werden? | 1 = überhaupt nicht wichtig; 2 = weniger wichtig; 3 = weder noch; 4 = etwas wichtig; 5 = sehr wichtig; 9 = weiß nicht/keine Angabe | How important do you think digitization will be for therapy and healthcare in the future? | 1 = very unimportant; 2 = somewhat unimportant; 3 = neither nor; 4 = somewhat important; 5 = very important; 9 = don't know/no answer |
| 3 | Wie wichtig, glauben Sie, wird die Digitalisierung in der Zukunft für die Förderung Ihrer Gesundheit werden? | 1 = überhaupt nicht wichtig; 2 = weniger wichtig; 3 = weder noch; 4 = etwas wichtig; 5 = sehr wichtig; 9 = weiß nicht/keine Angabe | How important do you think digitization will be for the promotion of your health in the future? | 1 = very unimportant; 2 = somewhat unimportant; 3 = neither nor; 4 = somewhat important; 5 = very important; 9 = don't know/no answer |
| 4 | Wie wichtig, glauben Sie, wird die Digitalisierung in der Zukunft für die Aufrechterhaltung Ihrer Gesundheit werden? | 1 = überhaupt nicht wichtig; 2 = weniger wichtig; 3 = weder noch; 4 = etwas wichtig; 5 = sehr wichtig; 9 = weiß nicht/keine Angabe | How important do you think digitization will be for the maintenance of your health in the future? | 1 = very unimportant; 2 = somewhat unimportant; 3 = neither nor; 4 = somewhat important; 5 = very important; 9 = don't know/no answer |
| 5 | Wie wahrscheinlich ist es, dass Sie sich in der Zukunft eine oder mehrere Apps aus dem Bereich der Prävention und Gesundheitsförderung auf Ihr Smartphone laden werden? | 1 = sehr unwahrscheinlich; 2 = eher unwahrscheinlich; 3 = noch offen; 4 = ziemlich wahrscheinlich; 5 = sehr wahrscheinlich; 9 = weiß nicht/keine Angabe | How likely is it that in the future you will download one or more apps from the field of prevention and health promotion onto your smartphone? | 1 = very unlikely; 2 = somewhat unlikely; 3 = unsure; 4 = somewhat likely; 5 = very likely; 9 = don't know/no answer |
| 6 | Wenn ich Gesundheitsapps nutze und installiere, schaue ich vorher, wer die App entwickelt und herausgegeben hat. | 1 = trifft überhaupt nicht zu; 2 = trifft eher nicht zu; 3 = unentschieden; 4 = trifft eher zu; 5 = trifft vollständig zu; 9 = weiß nicht/keine Angabe | When I use and install health apps, I check in advance who has developed and published the app. | 1 = strongly disagree; 2 = disagree; 3 = unsure; 4 = agree; 5 = strongly agree; 9 = don't know/no answer |
| 7 | Die Anzahl guter Bewertung ist ein wichtiges Kriterium für mich, wenn ich Gesundheitsapps nutze und installiere. | 1 = trifft überhaupt nicht zu; 2 = trifft eher nicht zu; 3 = unentschieden; 4 = trifft eher zu; 5 = trifft vollständig zu; 9 = weiß nicht/keine Angabe | The number of positive ratings is an important criterion for me when I use and install health apps. | 1 = strongly disagree; 2 = disagree; 3 = unsure; 4 = agree; 5 = strongly agree; 9 = don't know/no answer |
| 8 | Wenn ich ins Internet gehe, bin ich sehr darauf bedacht, keine persönlichen Angaben zu machen. | 1 = stimme überhaupt nicht zu; 2 = stimme weniger zu; 3 = stimme eher zu; 4 = stimme voll und ganz zu; 9 = weiß nicht/keine Angabe | When I go online, I take great care not to provide any personal information. | 1 = strongly disagree; 2 = disagree; 3 = agree; 4 = strongly agree; 9 = don't know/no answer |
| 9 | Wenn ich ins Internet gehe, bin ich besorgt, dass dies zu einer Verletzung meiner Privatsphäre führen könnte. | 1 = stimme überhaupt nicht zu; 2 = stimme weniger zu; 3 = stimme eher zu; 4 = stimme voll und ganz zu; 9 = weiß nicht/keine Angabe | When I go online, I am concerned that it could result in an invasion of my privacy. | 1 = strongly disagree; 2 = disagree; 3 = agree; 4 = strongly agree; 9 = don't know/no answer |
| 10 | Wie oft besuchen Sie an einem typischen Tag Social-Media-Plattformen, wie z. B. Facebook, Instagram, YouTube, WhatsApp etc.? Bitte geben Sie nur eine Antwort an. | 1 = Ich habe keinen Zugang zu einer Social-Media-Plattform *[weiter mit Frage 12]*; 2 = weniger als 5 Mal/Tag; 3 = 6-10 Mal/Tag; 4 = 11-20 Mal/Tag; 5 = 21-50 Mal/Tag; 6 = 51-100 Mal/Tag; 7 = mehr als 100 Mal/Tag; 9 = weiß nicht/keine Angabe | On a typical day, how often do you check social media platforms, such as Facebook, Instagram, YouTube, WhatsApp etc.? Please give only one answer. | 1 = I have no access to social media platforms *[go to Question 12]*; 2 = less than 5x/day; 3 = 6-10x/day; 4 = 11-20x/day; 5 = 21-50x/day; 6 = 51-100x/day; 7 = more than 100x/day; 9 = don't know/no answer |
| 11 | Welche ist die Social-Media-Plattform, die Sie am häufigsten Nutzen? Bitte geben Sie nur eine Antwort an. | 1 = soziale Netzwerke, wie z.B. Facebook, Twitter; 2 = Plattformen zur gemeinsamen Mediennutzung, wie z.B. YouTube, Instagram, Snapchat, Pinterest, TikTok; 3 = Plattformen für Sofortnachrichten, wie z. B. Facebook, Messenger, Viber, WhatsApp; 4 = sonstige; 9 = weiß nicht/keine Angabe | What is the social media platform that you use most often? Please give only one answer. | 1 = social networks, such as Facebook, Twitter; 2 = platforms for sharing, such as YouTube, Instagram, Snapchat, Pinterest, TikTok; 3 = platforms for instant messaging, such as Facebook, Messenger, Viber, WhatsApp; 4 = other; 9 = don't know/no answer |
| 12 | Angesichts der aktuellen Lage möchten wir von Ihnen erfahren, ob Sie das Internet nutzen, um sich über das Corona-Virus zu informieren und wie Sie diese Informationen nutzen. Wie oft stoßen Sie online auf Nachrichtenbeiträge über die aktuelle Corona-Krise, die Ihrer Meinung nach nicht ganz zutreffend sind? | 1 = oft; 2 = manchmal; 3 = selten; 4 = nie; 9 = weiß nicht/keine Angabe | In view of the current situation we would like to know whether you use the Internet to find out about the Coronavirus and how you use this information. How often do you come across online news on the COVID-19 pandemic that you think is not entirely accurate? | 1 = often; 2 = sometimes; 3 = seldom; 4 = never; 9 = don't know/no answer |
| 13 | Haben Sie jemals einen Nachrichtenbeitrag über die aktuelle Corona-Krise online geteilt, von dem Sie später herausfanden, dass er erfunden war? | 1 = ja; 2 = nein; 9 = weiß nicht/keine Angabe | Have you ever shared online news on the COVID-19 pandemic that you later found out was made up? | 1 = yes; 2 = no; 9 = don't know/no answer |
| 14 | Wie überzeugt sind Sie von Ihrer eigenen Fähigkeit, Nachrichtenbeiträge zu erkennen, die erfunden sind, z. B. Fake News? | 1 = sehr überzeugt; 2 = etwas überzeugt; 3 = nicht sehr überzeugt; 4 = überhaupt nicht überzeugt; 9 = weiß nicht/keine Angabe | How confident are you of your ability to recognise online news [on the COVID-19 pandemic] that is made up? | 1 = very confident; 2 = somewhat confident; 3 = somewhat not confident; 4 = not confident; 9 = don't know/no answer |
| 15 | Haben Sie die Corona-Warn-App des Robert Koch-Instituts auf Ihrem Smartphone installiert? | 1 = ja; 2 = nein; 9 = weiß nicht/keine Angabe | Have you installed the contact tracing app from the Robert Koch Institute [in Germany] on your smartphone? | 1 = yes; 2 = no; 9 = don't know/no answer |
| 16 | Nutzen Sie digitale Medien oder Software um körperlich aktiv zu sein, so dass sich Ihre Atemfrequenz erhöht? | 1 = nein, und ich habe nicht vor, damit anzufangen; 2 = nein, aber ich ziehe es in Betracht; 3 = nein, aber ich habe ernsthaft vor, damit anzufangen; 4 = ja, aber ich finde es ziemlich schwierig; 5 = ja und ich finde es ziemlich einfach; 9 = weiß nicht/keine Angabe *[wenn 1-3 oder 9 weiter mit Frage 21]* | Do you use digital media or software to be physically so active that your breathing rate increases? | 1 = no, and I don’t plan to start; 2 = no, but I consider to start; 3 = no, but I seriously intend to start; 4 = yes, but I find it quite difficult; 5 = yes and I find it very easy; 9 = don't know/no answer *[if 1-3 or 9 go to Question 21]* |
| 17 | Wie häufig sind Sie generell insgesamt 30 Minuten oder mehr so aktiv, dass sich Ihre Atemfrequenz erhöht? | 1 = täglich; 2 = mehrmals wöchentlich; 3 = einmal/Woche; 4 = weniger als einmal/Woche; 5 = nie; 9 = weiß nicht/keine Angabe | In general, how often are you so active for 30 minutes or longer that your breathing rate increases? | 1 = daily; 2 = several times/week; 3 = once a week; 4 = less than once a week; 5 = never; 9 = don't know/no answer |
| 18 | Wie häufig nutzen Sie digitale Medien oder Software, wie z. B. Freeletics, Gymondo oder YouTube Videos, um insgesamt 30 Minuten oder mehr so aktiv zu sein, dass sich Ihre Atemfrequenz erhöht? | 1 = täglich; 2 = mehrmals wöchentlich; 3 = einmal/Woche; 4 = weniger als einmal/Woche; 5 = nie; 9 = weiß nicht/keine Angabe | How often do you use digital media or software, such as Freeletics, Gymondo or YouTube videos for 30 minutes or longer so that your breathing rate increases? | 1 = daily; 2 = several times/week; 3 = once a week; 4 = less than once a week; 5 = never; 9 = don't know/no answer |
| 19 | Sind Sie sich sicher, dass Sie für insgesamt 30 Minuten oder mehr so aktiv sein können, dass sich Ihre Atemfrequenz erhöht? | 1 = stimme überhaupt nicht zu; 2 = stimme nicht zu; 3 = weder noch; 4 = stimme zu; 5 = stimme voll und ganz zu; 9 = weiß nicht/keine Angabe | Are you sure that you can be so active for 30 minutes or longer that your breathing rate increases? | 1 = strongly disagree; 2 = disagree; 3 = neither nor; 4 = agree; 5 = strongly agree; 9 = don't know/no response |
| 20 | Sind Sie sich sicher, dass Sie mit der Nutzung digitaler Medien oder Software, wie z. B. Freeletics, Gymondo oder YouTube Videos, für insgesamt 30 Minuten oder mehr so aktiv sein können, dass sich Ihre Atemfrequenz erhöht? | 1 = stimme überhaupt nicht zu; 2 = stimme nicht zu; 3 = weder noch; 4 = stimme zu; 5 = stimme voll und ganz zu; 9 = weiß nicht/keine Angabe | Are you sure that you can be so active with digital media or software, such as Freeletics, Gymondo or YouTube videos for 30 minutes or longer that your breathing rate increases? | 1 = strongly disagree; 2 = disagree; 3 = neither nor; 4 = agree; 5 = strongly agree; 9 = don't know/no response |
| 21 | Ich weiß, wie ich im Internet nützliche Gesundheitsinformationen finde (eHEALS 3). | 1 = stimme überhaupt nicht zu; 2 = stimme nicht zu; 3 = weder noch; 4 = stimme zu; 5 = stimme voll und ganz zu; 9 = weiß nicht/keine Angabe | I know how to find helpful health resources on the Internet (eHEALS 3). | 1 = strongly disagree; 2 = disagree; 3 = undecided; 4 = agree; 5 = strongly agree; 9 = don't know/no response |
| 22 | Ich weiß, wie ich das Internet nutzen kann, um Antworten auf meine Fragen rund um das Thema Gesundheit zu bekommen (eHEALS 4). | 1 = stimme überhaupt nicht zu; 2 = stimme nicht zu; 3 = weder noch; 4 = stimme zu; 5 = stimme voll und ganz zu; 9 = weiß nicht/keine Angabe | I know how to use the Internet to answer my questions about health (eHEALS 4). | 1 = strongly disagree; 2 = disagree; 3 = undecided; 4 = agree; 5 = strongly agree; 9 = don't know/no answer |
| 23 | Ich weiß, welche Quellen für Gesundheitsinformationen im Internet verfügbar sind (eHEALS 1). | 1 = stimme überhaupt nicht zu; 2 = stimme nicht zu; 3 = weder noch; 4 = stimme zu; 5 = stimme voll und ganz zu; 9 = weiß nicht/keine Angabe | I know what health resources are available on the Internet (eHEALS 1). | 1 = strongly disagree; 2 = disagree; 3 = undecided; 4 = agree; 5 = strongly agree; 9 = don't know/no answer |
| 24 | Ich weiß, wo im Internet ich nützliche Gesundheitsinformationen finden kann (eHEALS 2). | 1 = stimme überhaupt nicht zu; 2 = stimme nicht zu; 3 = weder noch; 4 = stimme zu; 5 = stimme voll und ganz zu; 9 = weiß nicht/keine Angabe | I know where to find helpful health resources on the Internet (eHEALS 2). | 1 = strongly disagree; 2 = disagree; 3 = undecided; 4 = agree; 5 = strongly agree; 9 = don't know/no answer |
| 25 | Ich weiß, wie ich Informationen aus dem Internet so nutzen kann, dass sie mir weiterhelfen (eHEALS 5). | 1 = stimme überhaupt nicht zu; 2 = stimme nicht zu; 3 = weder noch; 4 = stimme zu; 5 = stimme voll und ganz zu; 9 = weiß nicht/keine Angabe | I know how to use the health information I find on the Internet to help me (eHEALS 5). | 1 = strongly disagree; 2 = disagree; 3 = undecided; 4 = agree; 5 = strongly agree; 9 = don't know/no answer |
| 26 | Ich bin in der Lage, Informationen, die ich im Internet finde, kritisch zu bewerten (eHEALS 6). | 1 = stimme überhaupt nicht zu; 2 = stimme nicht zu; 3 = weder noch; 4 = stimme zu; 5 = stimme voll und ganz zu; 9 = weiß nicht/keine Angabe | I have the skills I need to evaluate the health resources I find on the Internet (eHEALS 6). | 1 = strongly disagree; 2 = disagree; 3 = undecided; 4 = agree; 5 = strongly agree; 9 = don't know/no answer |
| 27 | Ich kann im Internet zuverlässige von fragwürdigen Informationen unterscheiden (eHEALS 7). | 1 = stimme überhaupt nicht zu; 2 = stimme nicht zu; 3 = weder noch; 4 = stimme zu; 5 = stimme voll und ganz zu; 9 = weiß nicht/keine Angabe | I can tell high quality health resources from low quality health resources on the Internet (eHEALS 7). | 1 = strongly disagree; 2 = disagree; 3 = undecided; 4 = agree; 5 = strongly agree; 9 = don't know/no answer |
| 28 | Wenn ich gesundheitsbezogene Entscheidungen auf Basis von Informationen aus dem Internet treffe, fühle ich mich dabei sicher (eHEALS 8). | 1 = stimme überhaupt nicht zu; 2 = stimme nicht zu; 3 = weder noch; 4 = stimme zu; 5 = stimme voll und ganz zu; 9 = weiß nicht/keine Angabe | I feel confident in using information from the Internet to make health decisions (eHEALS 8). | 1 = strongly disagree; 2 = disagree; 3 = undecided; 4 = agree; 5 = strongly agree; 9 = don't know/no answer |

eHEALS, the eHealth Literacy Scale (the items in German adapted from Soellner R, Huber S, Reder M. The concept of eHealth literacy and its measurement. J Media Psychol. 2014;26(1):29-38. doi: 10.1027/1864-1105/a000104)

## Table S2. Participant characteristics (N=1014)

|  | **n** | **%** |
| --- | --- | --- |
| **Region by population** |  |  |
| under 2,000 | 20 | 2 |
| 2,000-5,000 | 30 | 3 |
| 5,000-20,000 | 73 | 7 |
| 20,000-50,000 | 108 | 11 |
| 50,000-100,000 | 109 | 11 |
| 100,000-500,000 | 282 | 28 |
| 500,000 or more | 392 | 39 |
| **Region by state** |  |  |
| former West-Germany | 829 | 82 |
| former East-Germany | 185 | 18 |
| **State** |  |  |
| 1. North Rhine-Westphalia | 176 | 17 |
| 1. Bavaria | 162 | 16 |
| 1. Baden-Wuerttemberg | 123 | 12 |
| 1. Lower Saxony | 112 | 11 |
| 1. Berlin11 | 68 | 7 |
| 1. Hesse | 68 | 7 |
| 1. Rhineland-Palatinate | 60 | 6 |
| 1. Saxony | 48 | 5 |
| 1. Brandenburg | 40 | 4 |
| 1. Schleswig-Holstein | 33 | 3 |
| 1. Saxony-Anhalt | 29 | 3 |
| 1. Hamburg | 25 | 2 |
| 1. Thuringia | 25 | 2 |
| 1. Mecklenburg-Vorpommern | 21 | 2 |
| 1. Bremen | 13 | 1 |
| 1. Saarland | 11 | 1 |

## Table S3. Digitization of health in the future (N=1014)

| **Q1. Do you use digital technologies for your health?** | | | | | |
| --- | --- | --- | --- | --- | --- |
|  | | Frequency | Percent | Valid Percent | Cumulative Percent |
| Valid | no | 431 | 42.5 | 42.5 | 42.5 |
|  | yes | 579 | 57.1 | 57.1 | 99.6 |
|  | I don‎‎'t know / No answer | 4 | .4 | .4 | 100.0 |
|  | Total | 1014 | 100.0 | 100.0 |  |
| **Q2A. How important do you think digitization will be for therapy and healthcare in the future?** | | | | | |
|  | | Frequency | Percent | Valid Percent | Cumulative Percent |
| Valid | very unimportant | 12 | 1.2 | 1.2 | 1.2 |
|  | somewhat unimportant | 50 | 4.9 | 4.9 | 6.1 |
|  | neither nor | 42 | 4.1 | 4.1 | 10.3 |
|  | somewhat important | 317 | 31.3 | 31.3 | 41.5 |
|  | very important | 581 | 57.3 | 57.3 | 98.8 |
|  | I don't know / No answer | 12 | 1.2 | 1.2 | 100.0 |
|  | Total | 1014 | 100.0 | 100.0 |  |
| **Q2B. How important do you think digitization will be for the promotion of your health in the future?** | | | | | |
|  | | Frequency | Percent | Valid Percent | Cumulative Percent |
| Valid | very unimportant | 47 | 4.6 | 4.6 | 4.6 |
|  | somewhat unimportant | 152 | 15.0 | 15.0 | 19.6 |
|  | neither nor | 89 | 8.8 | 8.8 | 28.4 |
|  | somewhat important | 364 | 35.9 | 35.9 | 64.3 |
|  | very important | 340 | 33.5 | 33.5 | 97.8 |
|  | I don't know / No answer | 22 | 2.2 | 2.2 | 100.0 |
|  | Total | 1014 | 100.0 | 100.0 |  |
| **Q2C. How important do you think digitization will be for the maintanance of your health in the future?** | | | | | |
|  | | Frequency | Percent | Valid Percent | Cumulative Percent |
| Valid | very unimportant | 60 | 5.9 | 5.9 | 5.9 |
|  | somewhat unimportant | 164 | 16.2 | 16.2 | 22.1 |
|  | neither nor | 95 | 9.4 | 9.4 | 31.5 |
|  | somewhat important | 332 | 32.7 | 32.7 | 64.2 |
|  | very important | 336 | 33.1 | 33.1 | 97.3 |
|  | I don't know / No answer | 27 | 2.7 | 2.7 | 100.0 |
|  | Total | 1014 | 100.0 | 100.0 |  |

## Table S4. Digitization, smartphone apps and Internet use (N=1014)

| **Q3. How likely is it that in the future you will download one or more apps from the field of prevention and health promotion onto your smartphone?** | | | | | | | | | | |
| --- | --- | --- | --- | --- | --- | --- | --- | --- | --- | --- |
|  | | | Frequency | | Percent | | Valid Percent | | Cumulative Percent | |
| Valid | | very unlikely | 241 | | 23.8 | | 23.8 | | 23.8 | |
|  |  | somewhat unlikely | 187 | | 18.4 | | 18.4 | | 42.2 | |
|  |  | neutral | 318 | | 31.4 | | 31.4 | | 73.6 | |
|  |  | somewhat likely | 149 | | 14.7 | | 14.7 | | 88.3 | |
|  |  | very likely | 111 | | 10.9 | | 10.9 | | 99.2 | |
|  |  | I don't know / No answer | 8 | | .8 | | .8 | | 100.0 | |
|  |  | Total | 1014 | | 100.0 | | 100.0 | |  | |
| **Q4A. When I use and install health apps. I check in advance who has developed and published the app.** | | | | | | | | | | |
|  | | | Frequency | | Percent | | Valid Percent | | Cumulative Percent | |
| Valid | | strongly disagree | 232 | | 22.9 | | 22.9 | | 22.9 | |
|  |  | disagree | 152 | | 15.0 | | 15.0 | | 37.9 | |
|  |  | unsure | 75 | | 7.4 | | 7.4 | | 45.3 | |
|  |  | agree | 206 | | 20.3 | | 20.3 | | 65.6 | |
|  |  | strongly agree | 301 | | 29.7 | | 29.7 | | 95.3 | |
|  |  | I don't know / No answer | 48 | | 4.7 | | 4.7 | | 100.0 | |
|  |  | Total | 1014 | | 100.0 | | 100.0 | |  | |
| **Q4B. The number of positive ratings is an important criterion for me when I use and install health apps.** | | | | | | | | | | |
|  | | | Frequency | | Percent | | Valid Percent | | Cumulative Percent | |
| Valid | | strongly disagree | 184 | | 18.1 | | 18.1 | | 18.1 | |
|  |  | disagree | 116 | | 11.4 | | 11.4 | | 29.6 | |
|  |  | unsure | 106 | | 10.5 | | 10.5 | | 40.0 | |
|  |  | agree | 323 | | 31.9 | | 31.9 | | 71.9 | |
|  |  | strongly agree | 235 | | 23.2 | | 23.2 | | 95.1 | |
|  |  | I don't know / No answer | 50 | | 4.9 | | 4.9 | | 100.0 | |
|  |  | Total | 1014 | | 100.0 | | 100.0 | |  | |
| **Q5A. When I go online, I take great care not to provide any personal information.** | | | | | | | | | | |
|  | | | Frequency | | Percent | | Valid Percent | | Cumulative Percent | |
| Valid | | strongly disagree | 35 | | 3.5 | | 3.5 | | 3.5 | |
|  |  | disagree | 176 | | 17.4 | | 17.4 | | 20.8 | |
|  |  | agree | 336 | | 33.1 | | 33.1 | | 53.9 | |
|  |  | strongly agree | 459 | | 45.3 | | 45.3 | | 99.2 | |
|  |  | I don't know / No answer | 8 | | .8 | | .8 | | 100.0 | |
|  |  | Total | 1014 | | 100.0 | | 100.0 | |  | |
| **Q5B. When I go online, I am concerned that it could result in an invasion of my privacy.** | | | | | | | | | | |
|  | | | Frequency | | Percent | | Valid Percent | | Cumulative Percent | |
| Valid | | strongly disagree | 87 | | 8.6 | | 8.6 | | 8.6 | |
|  |  | disagree | 296 | | 29.2 | | 29.2 | | 37.8 | |
|  |  | agree | 310 | | 30.6 | | 30.6 | | 68.3 | |
|  |  | strongly agree | 304 | | 30.0 | | 30.0 | | 98.3 | |
|  |  | I don't know / No answer | 17 | | 1.7 | | 1.7 | | 100.0 | |
|  |  | Total | 1014 | | 100.0 | | 100.0 | |  | |
| **Q6.On a typical day, how often do you check social media platforms, such as Facebook, Instagram, YouTube, WhatsApp etc.? Please give only one answer.** | | | | | | | | | | |
|  | | | | Frequency | | Percent | | Valid Percent | | Cumulative Percent |
| Valid | | I have no access to social media platforms | | 146 | | 14.4 | | 14.4 | | 14.4 |
|  |  | < 5x / day | | 334 | | 32.9 | | 32.9 | | 47.3 |
|  |  | 6-10x / day | | 259 | | 25.5 | | 25.5 | | 72.9 |
|  |  | 11-20x / day | | 139 | | 13.7 | | 13.7 | | 86.6 |
|  |  | 21-50x / day | | 96 | | 9.5 | | 9.5 | | 96.1 |
|  |  | 51-100x / day | | 24 | | 2.4 | | 2.4 | | 98.4 |
|  |  | > 100x / day | | 7 | | .7 | | .7 | | 99.1 |
|  |  | I don't know / No answer | | 9 | | .9 | | .9 | | 100.0 |
|  |  | Total | | 1014 | | 100.0 | | 100.0 | |  |
| **Q7. What is the social media platform that you use most often? Please give only one answer.** | | | | | | | | | | |
|  | | | | Frequency | | Percent | | Valid Percent | | Cumulative Percent |
| Valid | social networks, such as Facebook, Twitter | | | 98 | | 9.7 | | 11.3 | | 11.3 |
|  | platforms for sharing, such as YouTube, Instagram, Snapchat, Pinterest, TikTok | | | 181 | | 17.9 | | 20.9 | | 32.1 |
|  | platforms for instant messaging, such as Facebook, Messenger, Viber, WhatsApp | | | 560 | | 55.2 | | 64.5 | | 96.7 |
|  | other | | | 13 | | 1.3 | | 1.5 | | 98.2 |
|  | I don't know / No answer | | | 16 | | 1.6 | | 1.8 | | 100.0 |
|  | Total | | | 868 | | 85.6 | | 100.0 | |  |
| Missing | No access to social media | | | 146 | | 14.4 | |  | |  |
| Total | | | | 1014 | | 100.0 | |  | |  |

## Table S5. Digitization and COVID-19 pandemic (N=1014)

| **Q8. How often do you come across online news on the COVID-19 pandemic that you think are not entirely accurate?** | | | | | |
| --- | --- | --- | --- | --- | --- |
|  | | Frequency | Percent | Valid Percent | Cumulative Percent |
| Valid | often | 270 | 26.6 | 26.6 | 26.6 |
|  | sometimes | 262 | 25.8 | 25.8 | 52.5 |
|  | seldom | 272 | 26.8 | 26.8 | 79.3 |
|  | never | 171 | 16.9 | 16.9 | 96.2 |
|  | I don't know / No answer | 39 | 3.8 | 3.8 | 100.0 |
|  | Total | 1014 | 100.0 | 100.0 |  |
| **Q9. Have you ever shared online news on the COVID-19 pandemic that you later found out was made up?** | | | | | |
|  | | Frequency | Percent | Valid Percent | Cumulative Percent |
| Valid | yes | 56 | 5.5 | 5.5 | 5.5 |
|  | no | 948 | 93.5 | 93.5 | 99.0 |
|  | I don‎‎'t know / No answer | 10 | 1.0 | 1.0 | 100.0 |
|  | Total | 1014 | 100.0 | 100.0 |  |
| **Q10. How confident are you of your ability to recognise online news [on the COVID-19 pandemic] that is made up?** | | | | | |
|  | | Frequency | Percent | Valid Percent | Cumulative Percent |
| Valid | very confident | 349 | 34.4 | 34.4 | 34.4 |
|  | somewhat confident | 442 | 43.6 | 43.6 | 78.0 |
|  | somewhat not confident | 117 | 11.5 | 11.5 | 89.5 |
|  | not confident | 71 | 7.0 | 7.0 | 96.5 |
|  | I don't know / No answer | 35 | 3.5 | 3.5 | 100.0 |
|  | Total | 1014 | 100.0 | 100.0 |  |
| **Q11. Have you installed the contact tracing app from the Robert Koch Institute [in Germany] on your smartphone?** | | | | | |
|  | | Frequency | Percent | Valid Percent | Cumulative Percent |
| Valid | yes | 433 | 42.7 | 42.7 | 42.7 |
|  | no | 575 | 56.7 | 56.7 | 99.4 |
|  | I don‎‎'t know / No answer | 6 | .6 | .6 | 100.0 |
|  | Total | 1014 | 100.0 | 100.0 |  |

## Table S6. Digitization and physical activity (n=220)

| **Q12. Do you use digital media or software to be physically so active that your breathing rate increases?** | | | | | | | | | |
| --- | --- | --- | --- | --- | --- | --- | --- | --- | --- |
|  | | | | Frequency | Percent | | Valid Percent | Cumulative Percent | |
| Valid | | no, and I don’t plan to start | | 640 | 63.1 | | 63.1 | 63.1 | |
|  |  | no, but I consider to start | | 129 | 12.7 | | 12.7 | 75.8 | |
|  |  | no, but I seriously intend to start | | 12 | 1.2 | | 1.2 | 77.0 | |
|  |  | yes, but I find it quite difficult | | 33 | 3.3 | | 3.3 | 80.3 | |
|  |  | yes and I find it very easy | | 187 | 18.4 | | 18.4 | 98.7 | |
|  |  | I don‎‎'t know / No answer | | 13 | 1.3 | | 1.3 | 100.0 | |
|  |  | Total | | 1014 | 100.0 | | 100.0 |  | |
| **Q13A. In general, how often are you so active for 30 minutes or longer that your breathing rate increases?** | | | | | | | | | |
|  | | | | Frequency | Percent | | Valid Percent | Cumulative Percent | |
| Valid | daily | | | 53 | 5.2 | | 24.1 | 24.1 | |
|  | several times/week | | | 112 | 11.0 | | 50.9 | 75.0 | |
|  | once a week | | | 39 | 3.8 | | 17.7 | 92.7 | |
|  | less than once a week | | | 9 | .9 | | 4.1 | 96.8 | |
|  | never | | | 5 | .5 | | 2.3 | 99.1 | |
|  | I don‎‎'t know / No answer | | | 2 | .2 | | .9 | 100.0 | |
|  | Total | | | 220 | 21.7 | | 100.0 |  | |
| Missing | Nonuser | | | 794 | 78.3 | |  |  | |
| Total | | | | 1014 | 100.0 | |  |  | |
| **Q13B. How often do you use digital media or software, such as Freeletics, Gymondo or YouTube videos for 30 minutes or longer so that your breathing rate increases?** | | | | | | | | | |
|  | | | Frequency | | | Percent | Valid Percent | Cumulative Percent | |
| Valid | daily | | 20 | | | 2.0 | 9.1 | 9.1 | |
|  | several times/week | | 73 | | | 7.2 | 33.2 | 42.3 | |
|  | once a week | | 47 | | | 4.6 | 21.4 | 63.6 | |
|  | less than once a week | | 37 | | | 3.6 | 16.8 | 80.5 | |
|  | never | | 43 | | | 4.2 | 19.5 | 100.0 | |
|  | Total | | 220 | | | 21.7 | 100.0 |  | |
| Missing | Nonuser | | 794 | | | 78.3 |  |  | |
| Total | | | 1014 | | | 100.0 |  |  | |
| **Q14A. Are you sure that you can be so active for 30 minutes or longer that your breathing rate increases?** | | | | | | | | | |
|  | | | | Frequency | Percent | | Valid Percent | | Cumulative Percent |
| Valid | strongly disagree | | | 6 | .6 | | 2.7 | | 2.7 |
|  | disagree | | | 5 | .5 | | 2.3 | | 5.0 |
|  | neither nor | | | 14 | 1.4 | | 6.4 | | 11.4 |
|  | agree | | | 66 | 6.5 | | 30.0 | | 41.4 |
|  | strongly agree | | | 127 | 12.5 | | 57.7 | | 99.1 |
|  | I don't know / No answer | | | 2 | .2 | | .9 | | 100.0 |
|  | Total | | | 220 | 21.7 | | 100.0 | |  |
| Missing | Nonuser | | | 794 | 78.3 | |  | |  |
| Total | | | | 1014 | 100.0 | |  | |  |
| **Q14B. Are you sure that you can be so active with digital media or software, such as Freeletics, Gymondo or YouTube videos for 30 minutes or longer that your breathing rate increases?** | | | | | | | | | |
|  | | | | Frequency | Percent | | Valid Percent | | Cumulative Percent |
| Valid | strongly disagree | | | 8 | .8 | | 3.6 | | 3.6 |
|  | disagree | | | 16 | 1.6 | | 7.3 | | 10.9 |
|  | neither nor | | | 24 | 2.4 | | 10.9 | | 21.8 |
|  | agree | | | 71 | 7.0 | | 32.3 | | 54.1 |
|  | strongly agree | | | 98 | 9.7 | | 44.5 | | 98.6 |
|  | I don't know / No answer | | | 3 | .3 | | 1.4 | | 100.0 |
|  | Total | | | 220 | 21.7 | | 100.0 | |  |
| Missing | Nonuser | | | 794 | 78.3 | |  | |  |
| Total | | | | 1014 | 100.0 | |  | |  |

## Table S7. Digitization and perceived eHealth literacy: internal consistency (n=928)

| **Cronbach's Alpha = 0.881 (N of items = 8)** | | | | |
| --- | --- | --- | --- | --- |
| **eHEALS Item Statistics** | | Mean | Std. Deviation | N |
| 1. I know what health resources are available on the Internet (eHEALS 1). | | 3.80 | 1.012 | 928 |
| 2. I know where to find helpful health resources on the Internet (eHEALS 2). | | 3.94 | .974 | 928 |
| 3. I know how to find helpful health resources on the Internet (eHEALS 3). | | 3.97 | 1.076 | 928 |
| 4. I know how to use the Internet to answer my questions about health (eHEALS 4). | | 4.11 | .938 | 928 |
| 5. I know how to use the health information I find on the Internet to help me (eHEALS 5). | | 4.08 | .883 | 928 |
| 6. I have the skills I need to evaluate the health resources I find on the Internet (eHEALS 6).. | | 4.25 | .802 | 928 |
| 7. I can tell high quality health resources from low quality health resources on the Internet (eHEALS 7). | | 3.91 | .915 | 928 |
| 8. I feel confident in using information from the Internet to make health decisions (eHEALS 8). | | 3.11 | 1.101 | 928 |
| **eHEALS Item-Total Statistics** | **Scale Mean if Item Deleted** | **Scale Variance if Item Deleted** | **Corrected Item-Total Correlation** | **Cronbach's Alpha if Item Deleted** |
| 1. I know what health resources are available on the Internet (eHEALS 1). | 27.36 | 24.383 | .722 | .858 |
| 2. I know where to find helpful health resources on the Internet (eHEALS 2). | 27.22 | 24.393 | .756 | .854 |
| 3. I know how to find helpful health resources on the Internet (eHEALS 3). | 27.19 | 24.309 | .674 | .863 |
| 4. I know how to use the Internet to answer my questions about health (eHEALS 4). | 27.04 | 24.720 | .753 | .855 |
| 5. I know how to use the health information I find on the Internet to help me (eHEALS 5). | 27.08 | 25.714 | .683 | .863 |
| 6. I have the skills I need to evaluate the health resources I find on the Internet (eHEALS 6).. | 26.91 | 26.995 | .598 | .871 |
| 7. I can tell high quality health resources from low quality health resources on the Internet (eHEALS 7). | 27.24 | 26.694 | .538 | .876 |
| 8. I feel confident in using information from the Internet to make health decisions (eHEALS 8). | 28.05 | 25.987 | .483 | .885 |

eHEALS, the eHealth Literacy Scale

## Table S8. Digitization and perceived eHealth literacy: total sum score (n=928)

| eHEALS item | Valid n | Missing n | Mean | Median | Mode | SD | Min | Max |
| --- | --- | --- | --- | --- | --- | --- | --- | --- |
| 1. I know what health resources are available on the Internet (eHEALS 1). | 928 | 0 | 3.80 | 4.00 | 4 | 1.01 | 1 | 5 |
| 2. I know where to find helpful health resources on the Internet (eHEALS 2). | 928 | 0 | 3.94 | 4.00 | 4 | .97 | 1 | 5 |
| 3. I know how to find helpful health resources on the Internet (eHEALS 3). | 928 | 0 | 3.97 | 4.00 | 4 | 1.08 | 1 | 5 |
| 4. I know how to use the Internet to answer my questions about health (eHEALS 4). | 928 | 0 | 4.11 | 4.00 | 4 | .94 | 1 | 5 |
| 5. I know how to use the health information I find on the Internet to help me (eHEALS 5). | 928 | 0 | 4.08 | 4.00 | 4 | .88 | 1 | 5 |
| 6. I have the skills I need to evaluate the health resources I find on the Internet (eHEALS 6). | 928 | 0 | 4.25 | 4.00 | 4 | .80 | 1 | 5 |
| 7. I can tell high quality health resources from low quality health resources on the Internet (eHEALS 7). | 928 | 0 | 3.91 | 4.00 | 4 | .92 | 1 | 5 |
| 8. I feel confident in using information from the Internet to make health decisions (eHEALS 8). | 928 | 0 | 3.11 | 3.00 | 4 | 1.10 | 1 | 5 |
| **eHEALS sum score** | **928** | **0** | **31.16** | **32.00** | **32.00** | **5.71** | **8.00** | **40.00** |

eHEALS, the eHealth Literacy Scale

## Table S9. Digitization and perceived eHealth literacy: item statistics (n=928)

| **1. I know what health resources are available on the Internet (eHEALS 1).** | | | | | |
| --- | --- | --- | --- | --- | --- |
|  | | Frequency | Percent | Valid Percent | Cumulative Percent |
| Valid | strongly disagree | 28 | 3.0 | 3.0 | 3.0 |
|  | disagree | 99 | 10.7 | 10.7 | 13.7 |
|  | undecided | 122 | 13.1 | 13.1 | 26.8 |
|  | agree | 462 | 49.8 | 49.8 | 76.6 |
|  | strongly agree | 217 | 23.4 | 23.4 | 100.0 |
|  | Total | 928 | 100.0 | 100.0 |  |
| **2. I know where to find helpful health resources on the Internet (eHEALS 2).** | | | | | |
|  | | Frequency | Percent | Valid Percent | Cumulative Percent |
| Valid | strongly disagree | 31 | 3.3 | 3.3 | 3.3 |
|  | disagree | 71 | 7.7 | 7.7 | 11.0 |
|  | undecided | 72 | 7.8 | 7.8 | 18.8 |
|  | agree | 506 | 54.5 | 54.5 | 73.3 |
|  | strongly agree | 248 | 26.7 | 26.7 | 100.0 |
|  | Total | 928 | 100.0 | 100.0 |  |
| **3. I know how to find helpful health resources on the Internet (eHEALS 3).** | | | | | |
|  | | Frequency | Percent | Valid Percent | Cumulative Percent |
| Valid | strongly disagree | 43 | 4.6 | 4.6 | 4.6 |
|  | disagree | 72 | 7.8 | 7.8 | 12.4 |
|  | undecided | 79 | 8.5 | 8.5 | 20.9 |
|  | agree | 414 | 44.6 | 44.6 | 65.5 |
|  | strongly agree | 320 | 34.5 | 34.5 | 100.0 |
|  | Total | 928 | 100.0 | 100.0 |  |
| **4. I know how to use the Internet to answer my questions about health (eHEALS 4).** | | | | | |
|  | | Frequency | Percent | Valid Percent | Cumulative Percent |
| Valid | strongly disagree | 27 | 2.9 | 2.9 | 2.9 |
|  | disagree | 47 | 5.1 | 5.1 | 8.0 |
|  | undecided | 58 | 6.3 | 6.3 | 14.2 |
|  | agree | 458 | 49.4 | 49.4 | 63.6 |
|  | strongly agree | 338 | 36.4 | 36.4 | 100.0 |
|  | Total | 928 | 100.0 | 100.0 |  |
| **5. I know how to use the health information I find on the Internet to help me (eHEALS 5).** | | | | | |
|  | | Frequency | Percent | Valid Percent | Cumulative Percent |
| Valid | strongly disagree | 17 | 1.8 | 1.8 | 1.8 |
|  | disagree | 48 | 5.2 | 5.2 | 7.0 |
|  | undecided | 83 | 8.9 | 8.9 | 15.9 |
|  | agree | 479 | 51.6 | 51.6 | 67.6 |
|  | strongly agree | 301 | 32.4 | 32.4 | 100.0 |
|  | Total | 928 | 100.0 | 100.0 |  |
| **6. I have the skills I need to evaluate the health resources I find on the Internet (eHEALS 6).** | | | | | |
|  | | Frequency | Percent | Valid Percent | Cumulative Percent |
| Valid | strongly disagree | 13 | 1.4 | 1.4 | 1.4 |
|  | disagree | 31 | 3.3 | 3.3 | 4.7 |
|  | undecided | 41 | 4.4 | 4.4 | 9.2 |
|  | agree | 473 | 51.0 | 51.0 | 60.1 |
|  | strongly agree | 370 | 39.9 | 39.9 | 100.0 |
|  | Total | 928 | 100.0 | 100.0 |  |
| **7. I can tell high quality health resources from low quality health resources on the Internet (eHEALS 7).** | | | | | |
|  | | Frequency | Percent | Valid Percent | Cumulative Percent |
| Valid | strongly disagree | 19 | 2.0 | 2.0 | 2.0 |
|  | disagree | 67 | 7.2 | 7.2 | 9.3 |
|  | undecided | 118 | 12.7 | 12.7 | 22.0 |
|  | agree | 497 | 53.6 | 53.6 | 75.5 |
|  | strongly agree | 227 | 24.5 | 24.5 | 100.0 |
|  | Total | 928 | 100.0 | 100.0 |  |
| **8. I feel confident in using information from the Internet to make health decisions (eHEALS 8).** | | | | | |
|  | | Frequency | Percent | Valid Percent | Cumulative Percent |
| Valid | strongly disagree | 73 | 7.9 | 7.9 | 7.9 |
|  | disagree | 226 | 24.4 | 24.4 | 32.2 |
|  | undecided | 229 | 24.7 | 24.7 | 56.9 |
|  | agree | 328 | 35.3 | 35.3 | 92.2 |
|  | strongly agree | 72 | 7.8 | 7.8 | 100.0 |
|  | Total | 928 | 100.0 | 100.0 |  |

eHEALS, the eHealth Literacy Scale

## Table S10. Sociodemographic factors and digital technology use

| **Factor^a^** | **Use for health n=823^b^** | | | **Use for physical activity n=818^c^** | | | **Perceived eHealth literacy n=765^d^** | | |
| --- | --- | --- | --- | --- | --- | --- | --- | --- | --- |
|  | *B (SEM)* | *OR [95% CI]* | *P* | *B (SEM)* | *OR [95% CI]* | *P* | *B (SEM)* | *β* | *P* |
| gender | .25 (.14) | 1.28 [.96-1.70] | .087 | -.01 (.18) | .99 [.70-1.41] | .960 | .52 (.39) | .04 | .183 |
| age | -.01 (.01) | .99 [.98-1.00] | .132 | -.05 (.01) | **.95 [.94-.96]*** | <.001 | -.08 (.01) | **-.22*** | <.001 |
| education | -.06 (.07) | .95 [.82-1.09] | .450 | .20 (.09) | **1.22 [1.01-1.46]*** | .035 | .78 (.20) | **.14*** | <.001 |
| household income | .25 (.07) | **1.28 [1.11-1.47]*** | <.01 | .01 (.09) | 1.01 [.85-1.21] | .891 | 1.14 (.19) | **.21*** | <.001 |
| constant | -.22 (.47) |  | .633 | .29 (.56) |  | .611 | 28.13 (1.26) |  | <.001 |

The use of digital technologies for health was associated with higher household income (*OR*=1.28 [95% *CI*: 1.11-1.47]). The use of digital technologies for moderate physical activity was associated with younger age (*OR*=.95 [.94-.96]) and more education (*OR*=1.22 [1.01-1.46]). Higher perceived eHealth literacy was associated with younger age (*β*=-.22. *P*<.001), higher household income (*β*=.21. *P*<.001) and more education (*β*=.14. *P*<.001).

^a^Factor coding:

- gender: 1=male, 2=female;
- age in years measured on ascending scale from 14 to 93;
- education: 1=school pupil, 2=vocational college or basic secondary, 3=secondary without tertiary entrance qualification, 4=secondary with tertiary entrance qualification, 5=tertiary;
- household income (net/month): 1=under 1,500 Euro, 2=1,500 up to 2,500 Euro, 3=2,500 up to 3,500 Euro, 4=3,500 Euro or more

To avoid multicollinearity the household size (number of members) and the employment status were not included in the analysis because both variables affect the household income. Participants who did not report their household income were excluded from each analysis.

^b^Binary logistic regression. Dependent variable (use for health): no=0, yes=1; model *Χ^2^*=16.99, *df*=4, *P*=.002; Cox and Snell *R^2^*=.02

^c^Binary logistic regression. Dependent variable (use for physical activity): no=0, yes=1; model *Χ^2^*=77.24, *df=*4, *P*<.001; Cox and Snell *R^2^*=.09

^d^Multiple linear (ordinary least squares) regression. Dependent variable (perceived eHealth literacy): 8=lowest to 40=highest perceived eHealth literacy; model *F*=29.03, *df_model_=*4, *df_error_=*760, *P*<.001; adjusted *R^2^*=.13

**P*<.05

## Figure S1. Digitization of health in the future (N=1014; data weighted by region density, state, age, gender, employment, education and household size)

## Figure S2. Digitization, smartphone apps and Internet use (N=1014; data weighted by region density, state, age, gender, employment, education and household size)

## Figure S3. Digitization and COVID-19 pandemic (N=1014; data weighted by region density, state, age, gender, employment, education and household size)

|  |
| --- |
